# Acute Myocardial Infarction Detection Using Deep Learning-Enabled Electrocardiograms

**DOI:** 10.3389/fcvm.2021.654515

**Published:** 2021-08-24

**Authors:** Xiehui Chen, Wenqin Guo, Lingyue Zhao, Weichao Huang, Lili Wang, Aimei Sun, Lang Li, Fanrui Mo

**Affiliations:** ^1^Shenzhen Longhua District Central Hospital, Shenzhen, China; ^2^Department of Cardiology, Fuwai Hospital Chinese Academy of Medical Sciences, Shenzhen, Shenzhen, China; ^3^Department of Ambulatory Surgery, Huazhong University of Science and Technology Union Shenzhen Hospital, Shenzhen, China; ^4^Department of Cardiology, The First Affiliated Hospital of Guangxi Medical University, Nanning, China; ^5^Department of Cardiology, The Fourth Affiliated Hospital of Guangxi Medical University, Liuzhou, China

**Keywords:** acute myocardial infarction, deep learning, residual network, convolutional neural network, electrocardiogram

## Abstract

**Background:** Acute myocardial infarction (AMI) is associated with a poor prognosis. Therefore, accurate diagnosis and early intervention of the culprit lesion are of extreme importance. Therefore, we developed a neural network algorithm in this study to automatically diagnose AMI from 12-lead electrocardiograms (ECGs).

**Methods:** We used the open-source PTB-XL database as the training and validation sets, with a 7:3 sample size ratio. Twenty-One thousand, eight hundred thirty-seven clinical 12-lead ECGs from the PTB-XL dataset were available for training and validation (15,285 were used in the training set and 6,552 in the validation set). Additionally, we randomly selected 205 ECGs from a dataset built by Chapman University, CA, USA and Shaoxing People's Hospital, China, as the testing set. We used a residual network for training and validation. The model performance was experimentally verified in terms of area under the curve (AUC), precision, sensitivity, specificity, and F1 score.

**Results:** The AUC of the training, validation, and testing sets were 0.964 [95% confidence interval (CI): 0.961–0.966], 0.944 (95% CI: 0.939–0.949), and 0.977 (95% CI: 0.961–0.991), respectively. The precision, sensitivity, specificity, and F1 score of the deep learning model for AMI diagnosis from ECGs were 0.827, 0.824, 0.950, and 0.825, respectively, in the training set, 0.789, 0.818, 0.913, and 0.803, respectively, in the validation set, and 0.830, 0.951, 0.951, and 0.886, respectively, in the testing set. The AUC for automatic AMI location diagnosis of LMI, IMI, ASMI, AMI, ALMI were 0.969 (95% CI: 0.959–0.979), 0.973 (95% CI: 0.962–0.978), 0.987 (95% CI: 0.963–0.989), 0.961 (95% CI: 0.956–0.989), and 0.996 (95% CI: 0.957–0.997), respectively.

**Conclusions:** The residual network-based algorithm can effectively automatically diagnose AMI and MI location from 12-lead ECGs.

## Introduction

Acute myocardial infarction (AMI) is the main cause of mortality worldwide. The World Health Organization reported that ~15.9 million patients worldwide had an AMI in 2015, of which more than 3 million patients were diagnosed with acute ST-segment elevation myocardial infarction (1, 2). The mortality rate of patients with AMI is ~30%, with 50% of those deaths occurring before the patients arrive at the hospital (3). Early diagnosis and revascularization were associated with an improved prognosis for patients with AMI (4, 5). Therefore, early AMI recognition and culprit lesion intervention are important.

An electrocardiogram (ECG), with its low price, high safety, and fast reporting, is a routine examination for AMI diagnosis. The Fourth Universal Definition of Myocardial Infarction lists an ECG as an important element of AMI diagnosis (6). However, AMI diagnostic ability is limited in developing countries, where many patients with AMI are not diagnosed in time, which in turn delays treatment with revascularization. Physician workload and patient prognosis could be improved through deep learning algorithms that can automatically identify and diagnose AMI.

A deep learning algorithm can capture multiple features of an image and can automatically classify it (7). Deep learning has been widely used in medical fields, one such application is classifying 12-lead ECG results to automatically diagnose atrial fibrillation (8), hypertrophic cardiomyopathy (9), anemia (10), and other diseases. Therefore, the purpose of this study was to develop a convolutional neural network for automatically diagnosing AMI and evaluate the model's performance.

## Materials and Methods

### Data Collection

In this study, we used the open-access PTB-XL dataset as the training and validation sets (11). The PTB-XL dataset includes 21,837 clinical 12-lead ECGs from 18,885 patients. The length of each ECG signal is 10 s. Because 4,096 samples from the signal of each ECG lead to use as the neural network input, we used only the 500-Hz ECGs but not the 100-Hz ECGs as dataset. The AMI diagnoses were extracted from the file ptbxl_database.csv. Additionally, we used a dataset built by Chapman University, Orange, CA, USA, and Shaoxing People's Hospital, China, as the testing set (https://figshare.com/collections/ChapmanECG/4560497/2) (12). This dataset contains 12-lead ECGs from 10,646 patients. The length of each ECG signal is also 10 s and the ECG frequency is 100 Hz. There are 11 common heart rhythms and additional cardiovascular diseases in this dataset, with the images labeled by cardiovascular experts. The AMI diagnoses were extracted from the file Diagnostics.csv.

### Data Processing

Each ECG is a 12 ×5,000 matrix (12 leads, 10 s duration, 500 Hz sampling), where the first (12) represents the space dimension and the second (5, 000) represents the time dimension. We extracted 4,096 samples from the signal of each ECG lead to use as the neural network input. The original ECG data were pre-processed before training. To eliminate ECG signal baseline drift and low-power noise, we first used a low-pass filter on the original data to obtain a baseline and zeroed the average value to make the baseline flat. Next, we denoised the data by filtering the high-frequency signals.

### Data Splitting

Of the PTB-XL data, 30% were used for model validation and the remainder were used for model training. There were relatively few ECGs (only 41 cases) with an AMI diagnosis in the database from Chapman University and Shaoxing People's Hospital. Therefore, we randomly selected 164 non-AMI ECGs (four times the number of AMI ECGs) and merged them with the AMI ECGs to form the testing set (205 cases in total).

### Model Development

We used a residual network with a structure similar to that of a convolutional neural network (13). This architecture allows a deep neural network to be trained effectively by skipping connections. The network consisted of a convolutional layer (Conv) and four residual blocks, each of which had two convolutional layers. The output of the last block was fed back to a fully connected layer (dense) with a sigmoid activation function. The output of each convolutional layer was rescaled using batch normalization and fed into a rectified linear activation unit (ReLU). Dropout50 was applied after non-linearity. The filter length of the convolutional layer was 16. Starting from the first convolutional layer and residual block with 4,096 samples and 64 filters, 64 filters were added for each subsequent residual block, and four times subsampling was performed for each residual block. A convolutional layer with maxpooling51 and a filter length of 1 (1 ×1 Conv) was included in the skip connection to match the size of the main branch signal. The average cross-entropy was minimized using the Adam optimizer 52 with default parameters and a learning rate (*lr*) of 0.001. When there was no improvement within seven consecutive iterations when verifying the loss, *lr* was reduced by a factor of ten. The initial value of the neural network weight was as referenced in the literature (14), and the initial bias was 0. In the optimization process, the final model was the one with the best verification results after 40 epochs.

Because neural networks are initialized randomly, different initializations usually produce different results. To show the stability of the algorithm, we trained ten neural networks. The hyperparameters of these neural networks were the same, but the initializations were different. We chose the model in which the micro-average accuracy was immediately above the median.

Furthermore, we performed an additional analysis to evaluate the deep learning model performance in predicting the MI location. The number of ECG per MI location in the Chapman University and Shaoxing People's Hospital dataset was small; therefore, we did not use this dataset as the testing set in this analysis. Alternatively, we used 10% of the ECGs in the PTB-XL dataset as a testing set; the sample size ratio of the training and validation sets was 7:3 in the remaining ECGs. The deep learning model performance was evaluated in identifying five MI locations, namely, lateral myocardial infarction (LMI), inferior myocardial infarction (IMI), anteroseptal myocardial infarction (ASMI), anterior myocardial infarction (AMI), and anterolateral myocardial infarction (ALMI). Because the number of posterior MI (PMI) locations in the PTB-XL dataset was very small (only 17 cases), and because this location is commonly identified in 18-lead ECGs, we did not include it in the target identification object.

### Statistical Analysis

Continuous variables are presented as mean ± standard deviation, and categorical variables are presented as numbers (percentages). Differences in baseline variables between groups were ascertained using analysis of variance or the Kruskal-Wallis test for continuous variables and the chi-squared test or Fisher's exact test for dichotomous variables. We created a receiver operating characteristic (ROC) curve for the training, validation, and test sets and used the area under the curve (AUC) to evaluate the deep learning model performance in diagnosing AMI (15). Additional indicators for evaluating model performance include precision, specificity, sensitivity, and F1 score. Additionally, we performed the adjusted ROC curve to evaluate the influence of general metadata on the AUC. The statistical analysis was performed using R software version 3.5.2.

## Results

**Figure 1:**
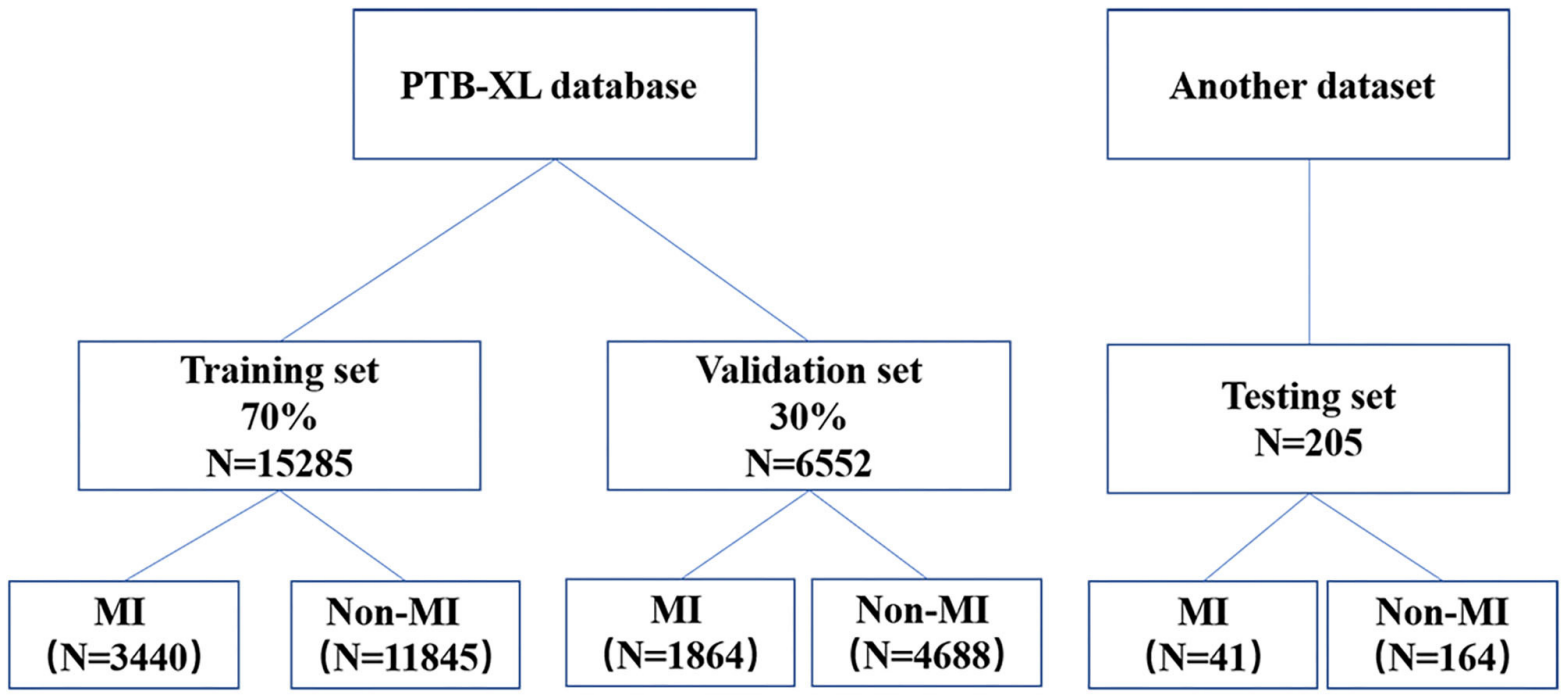
A flow diagram indicating the selection of electrocardiography for the training, validation, and testing.

**Figure 2:**
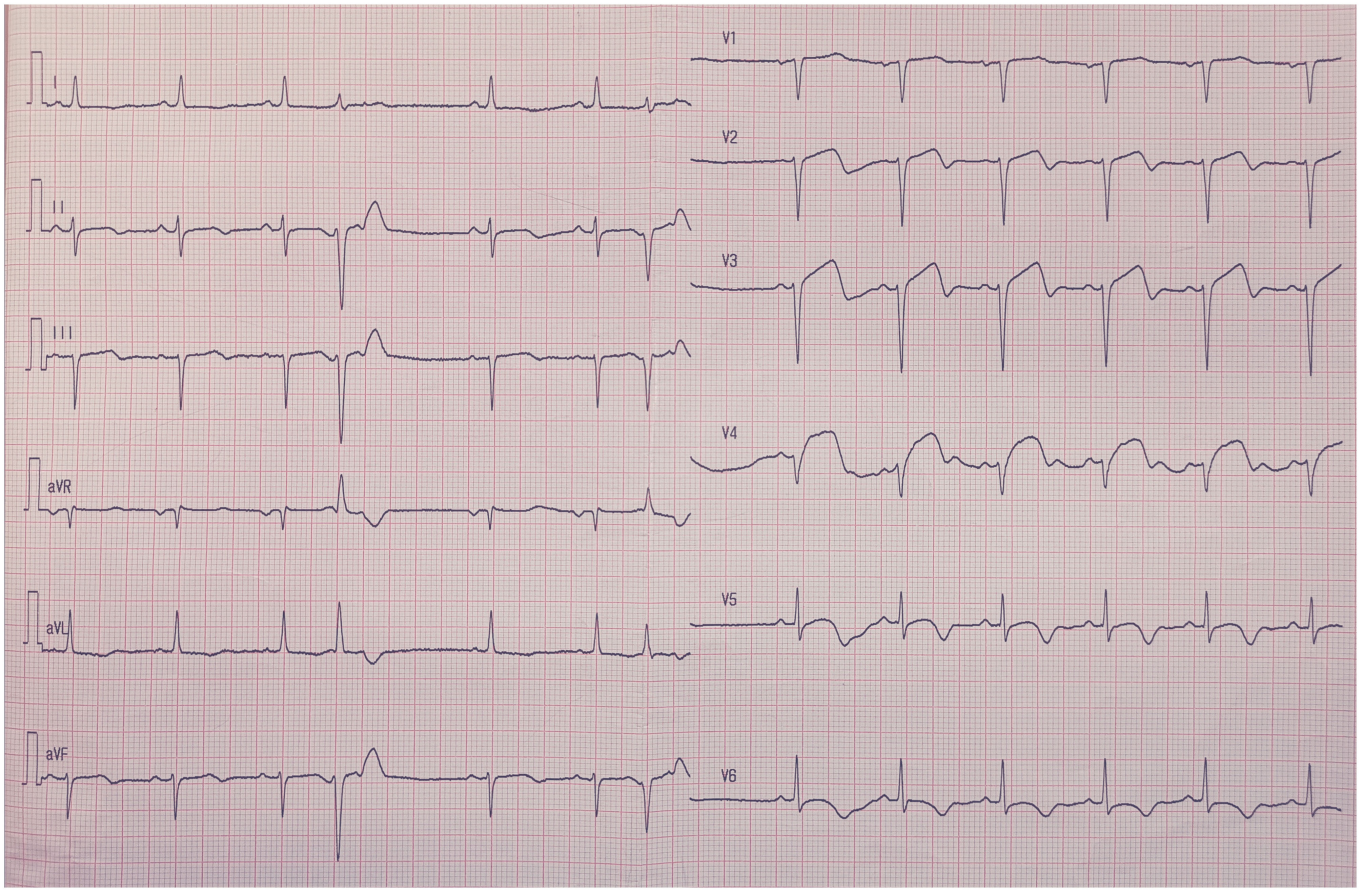
The electrocardiography feature of acute myocardial infarction. ST-segment elevation in two adjacent leads (V3–V4), with r-wave/Q-wave formation, and inverted or bidirectional T waves.

The sample size of the training, validation, and testing sets and the numbers of AMI ECGs and non-AMI ECGs are shown in Figure 1. There were 15,285 ECGs in the training set, of which 3,440 were AMI ECGs and 11,845 were non-AMI ECGs. There were 6,552 ECGs in the validation set, of which 1,864 were AMI ECGs and 4,688 were non-AMI ECGs. Finally, there were 205 ECGs in the testing set, of which 41 were AMI ECGs and 164 were non-AMI ECGs. The electrocardiogram criteria for AMI diagnosis is based on the 2012 joint ESC/ACCF/AHA/WHF Task Force for the Universal Definition of Myocardial Infarction, i.e., the new ST-segment elevation at the J point in two anatomically adjacent leads (16). For example, Figure 2 shows AMI ECG features with ST-segment elevation in two adjacent leads (V3–V4), with r-wave/Q-wave formation, and inverted or bidirectional T waves.

### Baseline Characteristics

The baseline characteristics between patients with AMI ECGs and those with non-AMI ECGs are shown in Table 1. In the training set, patients with AMI ECGs were associated with higher age, height, and weight and were less likely to be male (*P* < 0.05). In the validation set, patients with AMI ECGs were also associated with higher age, height, and weight and were less likely to be male (*P* < 0.05). In the testing set, patients with AMI ECGs were associated with higher ages than those with non-AMI ECGs (*P* < 0.05), but gender makeup was similar between the two groups (*P* = 0.403).

**Table 1:** The baseline characteristics between patients with acute myocardial infarction ECGs and those with non-myocardial infarction ECGs.

	**Non-MI**	**MI**	***P***
**Training set**		
Number	11,845	3,440	
Age [mean (SD)]	57.41 (17.98)	67.30 (13.00)	<0.001
Male (%)	6,190 (52.3)	1,354 (39.4)	<0.001
Height [mean (SD)]	167.18 (10.58)	168.16 (11.02)	<0.001
Weight [mean (SD)]	71.08 (15.79)	72.22 (16.61)	<0.001
**Validation set**		
Number	4,688	1,864	
Age [mean (SD)]	58.37 (16.41)	65.34 (12.34)	<0.001
Male (%)	2,304 (49.1)	610 (32.7)	<0.001
Height [mean (SD)]	168.39 (10.13)	168.97 (9.91)	0.038
Weight [mean (SD)]	72.15 (16.71)	73.75 (15.69)	<0.001
**Testing set**		
Number	164	41	
Age [mean (SD)]	58.87 (18.05)	67.83 (16.64)	0.004
Male (%)	102 (62.2)	29 (70.7)	0.403

*MI, myocardial infarction*.

### Performance of the Deep Learning Model

**Figure 3:**
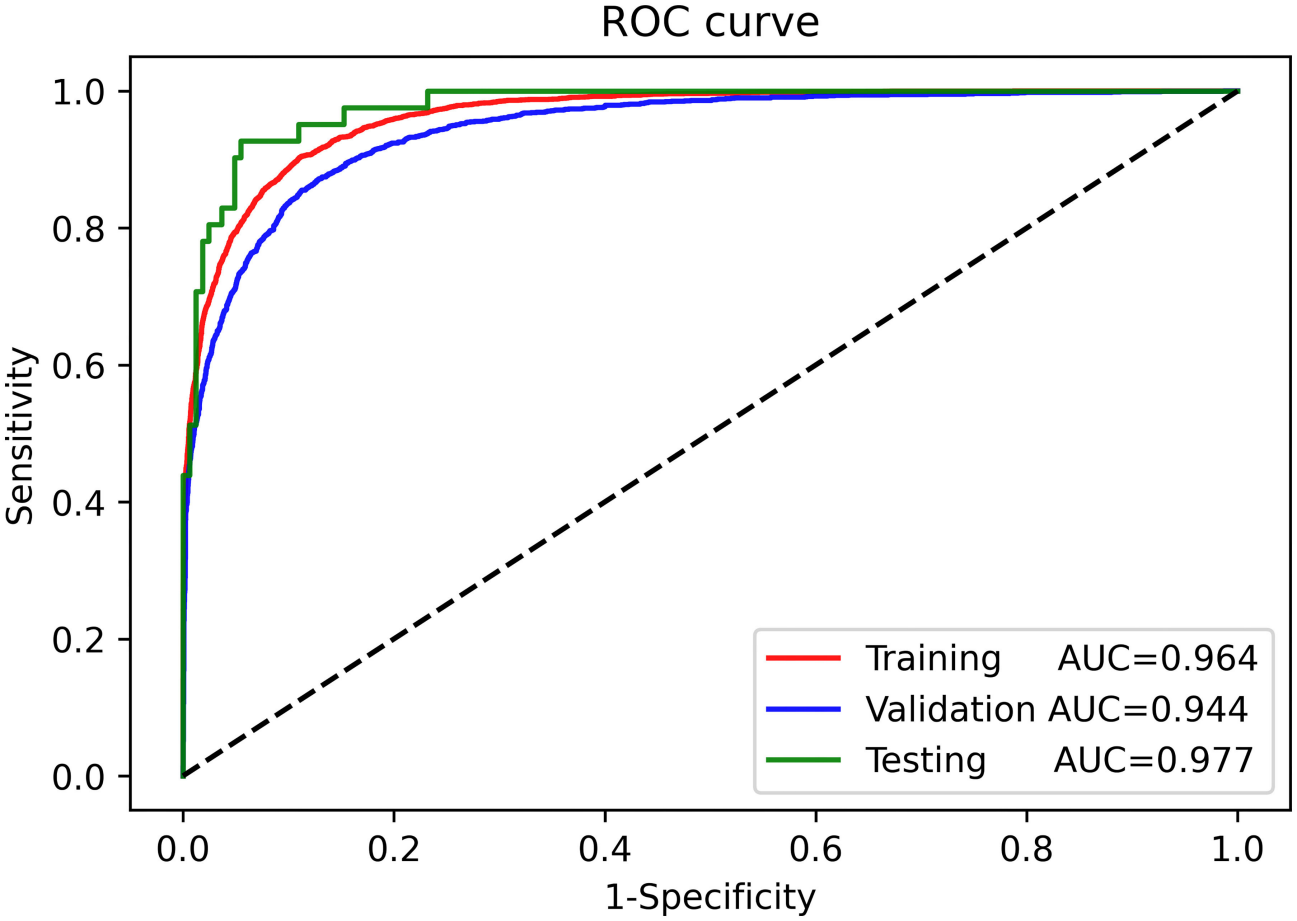
Receiver-operating characteristic curves for automatic diagnosis of acute myocardial infarction in the training, validation, and testing sets.

The ROC curves for automatic AMI diagnosis in the training, validation, and testing sets are shown in Figure 3. The AUC of the training, validation, and testing sets were 0.964 [95% confidence interval (CI): 0.961–0.966], 0.944 (95% CI: 0.939–0.949), and 0.977 (95% CI: 0.961–0.991), respectively. The adjusted AUC of the training, validation, and testing sets were 0.936, 0.908, 0.927, respectively. Table 2 shows the precision, sensitivity, specificity, and F1 scores of the deep learning model on the different datasets. For AMI ECGs, the precision, sensitivity, specificity, and F1 score were 0.827, 0.824, 0.950, and 0.825, respectively, when using the training set, 0.789, 0.818 0.913, and 0.803, respectively, when using the validation set, and 0.830, 0.951, 0.951, and 0.886, respectively, when using the testing set. For non-AMI ECGs, the precision, sensitivity, specificity, and F1 score were 0.943, 0.958, 0.799, and 0.950, respectively, using the training set, 0.887, 0.963, 0.692, and 0.923, respectively, when using the validation set, and 0.964, 0.982, 0.854, and 0.973, respectively, when using the testing set.

**Table 2:** The precision, sensitivity, specificity, and F1 scores of the deep learning model on the different datasets.

	**Precision**	**Sensitivity**	**Specificity**	**F1 score**
**Training dataset**				
MI	0.827	0.824	0.950	0.825
Non-MI	0.943	0.958	0.799	0.950
**Validation dataset**				
MI	0.789	0.818	0.913	0.803
Non-MI	0.887	0.963	0.692	0.923
**Testing dataset**				
MI	0.830	0.951	0.951	0.886
Non-MI	0.964	0.982	0.854	0.973

*MI, myocardial infarction*.

The number of ECGs diagnosed with false-negative results was 339 and 2 in the validation and testing set, respectively. The MI location of these ECGs is shown in Supplementary Table 1. These results show that most IML ECGs (191 cases) were not correctly identified by the deep learning model.

The ROC curves for automatic AMI location diagnosis are shown in Supplementary Figure 1. The AUC for automatic AMI location diagnosis of LMI, IMI, ASMI, AMI, ALMI were 0.969 (95% CI: 0.959–0.979), 0.973 (95% CI: 0.962–0.978), 0.987 (95% CI: 0.963–0.989), 0.961 (95% CI: 0.956–0.989), and 0.996 (95% CI: 0.957–0.997). The adjusted AUC for automatic AMI location diagnosis of LMI, IMI, ASMI, AMI, ALMI were 0.902, 0.934, 0.964, 0.866, and 0.958, respectively. The deep learning model performance in predicting the MI location in the testing set is shown in Supplementary Table 2. These results show that the precision, sensitivity, specificity, and F1 score of the model to predict the LMI location was 0.835, 0.786, 0.989, and 0.81, respectively. The precision, sensitivity, specificity, and F1 score of the model were 0.815, 0.892, 0.919, and 0.852, respectively, when predicting the IMI location, 0.923, 0.909, 0.982, and 0.916, respectively, when predicting the ASMI location, 0.520, 0.600, 0.987, and 0.557, respectively, when predicting the AMI location, and 0.932, 0.873, 0.998, and 0.902, respectively, when predicting the AlMI location.

## Discussion

A total of 10 neural networks were trained. These neural networks used the same hyperparameters but different initializations. The model with micro-average accuracy immediately above the median was selected. We used the training, validation, and testing sets to evaluate the model performance. These results show that the diagnostic performance of the residual network was satisfactory.

ECGs are associated with high specificity in AMI diagnosis and are therefore important for diagnosis. The AMI ECG features are ST-segment elevation in two adjacent leads, with or without Q-wave formation, and bidirectional or inverted T waves. Certain patients with chest pain were diagnosed with acute myocarditis, which resembles AMI because of its similar ECG features (17). Therefore, correct identification and rapid diagnosis of AMI will allow active treatment to begin (e.g., coronary intervention therapy) to save most of the necrotic myocardium and improve patient prognosis (18).

Additionally, many hospitals are equipped with ECG machines because they are convenient to use, and the reports can be obtained quickly. However, AMI diagnosis is still difficult because of physician shortage and poor diagnosis ability in developing countries. Therefore, the misdiagnosis rate and physician workload could be reduced by using deep learning to detect AMI automatically.

Gupta et al. developed a ConvNetQuake neural network model to correctly diagnose AMI from ECGs with a classification accuracy of 99.43%. Furthermore, their results also showed that the II, Vz, and V6 lead signals are important to correctly identify AMI (19). Acharya et al. developed a convolutional neural network model and used the II lead signal as the input with an average diagnostic accuracy of 93.53% (20). Jafarian et al. used an end-to-end neural network to automatically detect AMI with an accuracy of 98% (21). However, these studies used the PTB database as the training set and had no external validation. The PTB dataset contains only 549 records from 290 subjects (22). Therefore, the diagnostic performance of those models still needs to be validated using clinical data. Moreover, the ECG in the above-mentioned studies was spilt into multiple heartbeat to extend the dataset. In this study, we proposed a residual network-based algorithm to automatically diagnose AMI. The residual neural network uses a skip connection for the degradation phenomenon. Additionally, the residual network eliminates the problem of increasing layers in the neural network, which causes training difficulties such as gradient explosion and disappearance (13). Furthermore, this study uses the open-source PTB-XL dataset as the training and validation set (11). The PTB-XL dataset contains a large number of ECG signals, and a significant proportion of the ECGs show AMI, which improves the model's ability to identify AMI. Instead of the beat-to-beat classification used in previous studies (18–20), our study is based on 12-lead ECG exams, which are more common in clinical practice. In addition, we used a database built by Chapman University and Shaoxing People's Hospital as an external testing set (12), which fully confirms the superior performance of the residual network using different datasets. Identifying the AMI location is important in clinical practice because the treatment strategy might be different for different MI locations. For example, capacity management is different for patients with acute anterior AMI that for patients with acute right ventricular AMI (23). Accordingly, the proposed deep learning model performance in predicting the MI location was also evaluated. The results showed that the deep learning model can effectively automatically diagnose the MI location from 12-lead ECGs.

## Limitations

Despite its many advantages, this study has its limitations. First, our study was based on open-access ECG databases. These datasets included general metadata, such as age, sex, weight, and height, but other clinical features, such as the target vessel and clinical comorbidities, which might influence the AMI ECG feature, are not included. We performed the adjusted ROC curve to evaluate the influence of general metadata on the AUC. The results showed that AUC was robust to the general metadata. Second, our results need to be transformed into applications in the future, such as developing a program to be implanted in the ECG equipment to guide clinical practice. Third, certain patients with AMI have multivessel disease. Therefore, an algorithm needs to be developed to identify patients with such conditions. Fourth, the ECG and troponin should be comprehensively evaluated in the diagnosis of non-ST-elevation acute coronary syndrome (NSTE-ACS). NSTE-ACS diagnosis based on ECG results alone is still a challenge in clinical practice. Moreover, our algorithm is not suitable for NSTE-ACS patients. Thus, it is necessary to further develop deep learning algorithms for NSTE-ACS diagnosis in the future. Fifth, deep learning models are being increasingly used in medical fields. In our study, we mainly evaluated the deep learning model performance to automatically identify AMI and found that it could effectively automatically diagnose AMI from 12-lead ECGs. The software for automatic ECG interpretation is common and already available in daily practice. However, these software are mainly based on the expert system, Bayesian paradigm, and cluster analysis (24–26). A performance comparison between the deep learning model and the traditional paradigm is warranted in future work. Sixth, although our algorithm has great performance in automatically diagnosing MI location, it still has low accuracy and sensitivity in diagnosing AMI. Therefore, the results of this study should be interpreted with caution in clinical practice. Finally, AMI suspicion is mainly based on symptoms and medical history, which could not be covered by a better ECG interpretation.

## Conclusion

Based on the obtained results, it is concluded that the residual network-based algorithm developed in this study can effectively automatically diagnose AMI and MI location from 12-lead ECGs.

## Data Availability Statement

Publicly available datasets were used in this study. This data can be found at: https://figshare.com/collections/ChapmanECG/4560497/2 and https://physionet.org/content/ptb-xl/1.0.1/.

## Ethics Statement

The studies involving human participants were reviewed and approved by Research Ethics Committee of the Fuwai Hospital Chinese Academy of Medical Sciences, Shenzhen. Written informed consent for participation was not required for this study in accordance with the national legislation and the institutional requirements.

## Author Contributions

WG, XC, and FM designed the study and developed the neural network. LZ and AS performed the statistical analysis. WG wrote the manuscript. WH, XC, LW, and LL critically reviewed the manuscript. All authors contributed to the article and approved the final version.

## Conflict of Interest

The authors declare that the research was conducted in the absence of any commercial or financial relationships that could be construed as a potential conflict of interest.

## Supplementary Material

Supplementary Figure 1 The Receiver-operating characteristic curves for automatic acute myocardial infarction location diagnosis in the testing set.

Supplementary Table 1 The ECGs diagnosed with false-negative results in the validation and testing set.

Supplementary Table 2 The deep learning model performance in predicting the MI location in the testing set.
