# Corrigendum: Acute Myocardial Infarction Detection Using Deep Learning-Enabled Electrocardiograms

**DOI:** 10.3389/fcvm.2021.735864

**Published:** 2021-08-24

**Authors:** Xiehui Chen, Wenqin Guo, Lingyue Zhao, Weichao Huang, Lili Wang, Aimei Sun, Lang Li, Fanrui Mo

**Affiliations:** ^1^Shenzhen Longhua District Central Hospital, Shenzhen, China; ^2^Department of Cardiology, Fuwai Hospital Chinese Academy of Medical Sciences, Shenzhen, Shenzhen, China; ^3^Department of Ambulatory Surgery, Huazhong University of Science and Technology Union Shenzhen Hospital, Shenzhen, China; ^4^Department of Cardiology, The First Affiliated Hospital of Guangxi Medical University, Nanning, China; ^5^Department of Cardiology, The Fourth Affiliated Hospital of Guangxi Medical University, Liuzhou, China

**Keywords:** acute myocardial infarction, deep learning, residual network, convolutional neural network, electrocardiogram

In the original article, an author name was incorrectly spelled as Fangrui Mo. The correct spelling is Fanrui Mo.

In the original article, there were some errors in the affiliation list. For affiliation 2, instead of “Department of Cardiology, Fuwai Hospital Chinese Academy of Medical Sciences, Shenzhen, China”, it should be “Department of Cardiology, Fuwai Hospital Chinese Academy of Medical Sciences, Shenzhen, Shenzhen, China”.

For affiliation 5, instead of “Department of Cardiology, The Forth Affiliated Hospital of Guangxi Medical University, Liuzhou, China”, it should be “Department of Cardiology, The Fourth Affiliated Hospital of Guangxi Medical University, Liuzhou, China”.

Further, Dr. Xiehui Chen should also be listed as a corresponding author.

The authors apologize for these errors and state that this does not change the scientific conclusions of the article in any way. The original article has been updated.

## Publisher's Note

All claims expressed in this article are solely those of the authors and do not necessarily represent those of their affiliated organizations, or those of the publisher, the editors and the reviewers. Any product that may be evaluated in this article, or claim that may be made by its manufacturer, is not guaranteed or endorsed by the publisher.

