# Supplementary figures and images for: Acute Myocardial Infarction Detection Using Deep Learning-Enabled Electrocardiograms
Source: Front Cardiovasc Med. 2021 Aug 24;8:654515. doi: 10.3389/fcvm.2021.654515 (PMC8273385; doi:10.3389/fcvm.2021.654515)

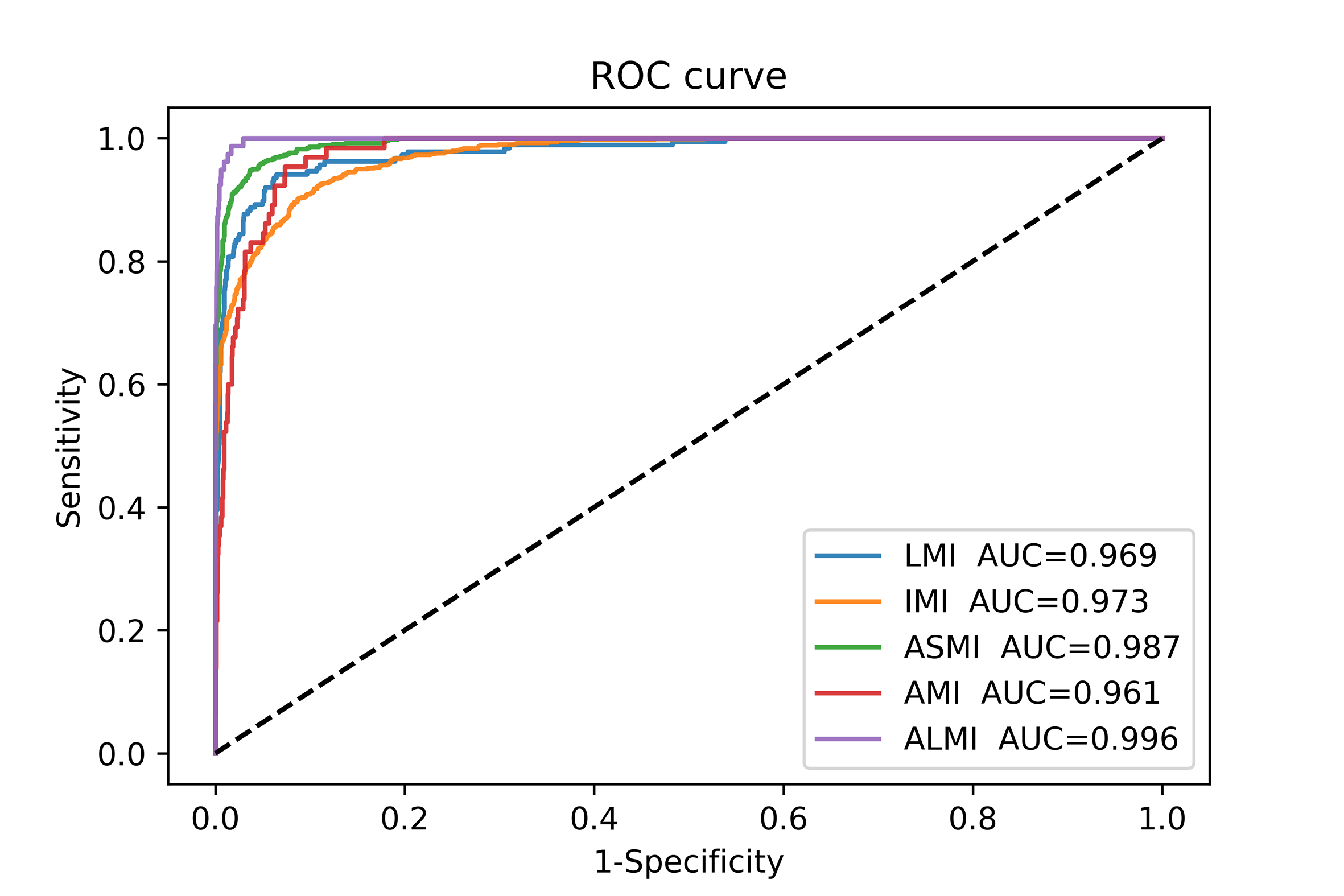

Supplement: Supplementary Figure 1 — The Receiver-operating characteristic curves for automatic acute myocardial infarction location diagnosis in the testing set. [file Image_1.TIFF]
