# Supplementary material for: Acute Myocardial Infarction Detection Using Deep Learning-Enabled Electrocardiograms
Source: Front Cardiovasc Med. 2021 Aug 24;8:654515. doi: 10.3389/fcvm.2021.654515 (PMC8273385; doi:10.3389/fcvm.2021.654515)
Supplement: Supplementary Table 1 — The ECGs diagnosed with false-negative results in the validation and testing set. [file Table_1.docx]

| **Supplemental Table 1.** The ECGs diagnosed with false-negative results in the validation and testing set. | | | | |
| --- | --- | --- | --- | --- |
| **Validation set**  **(N=339)** | | | **Testing set**  **(N=2)** | |
| **MI location** | | **Number** | **MI location** | **Number** |
| IMI |  | 191 | LMI | 1 |
| ASMI |  | 43 | IMI | 1 |
| LMI |  | 31 |  |  |
| ALMI |  | 19 |  |  |
| AMI |  | 19 |  |  |
| ILMI |  | 11 |  |  |
| ASMI | IMI | 6 |  |  |
| IPLMI |  | 4 |  |  |
| AMI | IMI | 4 |  |  |
| PMI |  | 3 |  |  |
| ASMI | LMI | 3 |  |  |
| ASMI | ALMI | 2 |  |  |
| ASMI | IPLMI | 1 |  |  |
| ASMI | ILMI | 1 |  |  |
| IMI | LMI | 1 |  |  |

IMI: Inferior myocardial infarction; ASMI: Anteroseptal myocardial infarction; LMI: Lateral myocardial infarction; ALMI: Anterolateral myocardial infarction; AMI: Anterior myocardial infarction; PMI: Posterior myocardial infarction; ILMI: Inferolateral myocardial infarction; IPLMI: Inferoposterolateral myocardial infarction.
