# Supplementary material for: Acute Myocardial Infarction Detection Using Deep Learning-Enabled Electrocardiograms
Source: Front Cardiovasc Med. 2021 Aug 24;8:654515. doi: 10.3389/fcvm.2021.654515 (PMC8273385; doi:10.3389/fcvm.2021.654515)
Supplement: Supplementary Table 2 — The deep learning model performance in predicting the MI location in the testing set. [file Table_2.docx]

| **Supplemental Table 2. The deep learning model performance in predicting the MI location in the testing set.** | | | | |
| --- | --- | --- | --- | --- |
| **MI location** | **Precision** | **Sensitivity** | **Specificity** | **F1 score** |
| LMI | 0.835 | 0.786 | 0.989 | 0.81 |
| IMI | 0.815 | 0.892 | 0.919 | 0.852 |
| ASMI | 0.923 | 0.909 | 0.982 | 0.916 |
| AMI | 0.52 | 0.6 | 0.987 | 0.557 |
| ALMI | 0.932 | 0.873 | 0.998 | 0.902 |
| LMI: Lateral myocardial infarction; IMI: Inferior myocardial infarction; ASMI: Anteroseptal myocardial infarction; AMI: Anterior myocardial infarction; ALMI: Anterolateral myocardial infarction. | | | | |
